# INFLUENCES OF HAVING COGNITIVE IMPAIRMENT OR DEMENTIA ON SOCIAL ENGAGEMENT AMONG OLDER ADULTS: A SYSTEMATIC REVIEW

**DOI:** 10.1093/geroni/igad104.1565

**Published:** 2023-12-21

**Authors:** Clara Scher, Takashi Amano, Addam Reynolds, Yuane Jia

**Affiliations:** Rutgers, The State University of New Jersey, New Brunswick, New Jersey, United States; Rutgers University, Newark, New Jersey, United States; University of Southern California, Los Angeles, California, United States; Rutgers University, Newark, New Jersey, United States

## Abstract

Studies have suggested that promoting and maintaining social engagement are positively associated with various well-being outcomes in later life. One notable barrier to sustaining social engagement in later life is development of cognitive impairment (CI) and/or Alzheimer’s disease and related dementias (ADRD). This study aims to synthesize existing literature exploring the impact of CI and ADRD on social engagement among older adults. We conducted a search of studies written in English language in the following databases: PsychInfo, PubMed, MEDLINE, CINAHL, and The Cochrane Library. Inclusion criteria included: observational studies that were conducted outside of residential long-term care facilities, included social engagement as an outcome variable, and examined the impact of cognitive impairment and/or ADRD on social engagement. Eighteen studies were included for qualitative synthesis. The majority of studies used author-developed scales to measure social engagement. Three utilized formal clinical diagnosis to measure cognitive impairment and ADRD. Twenty unique social activities were included, such as volunteering, attending community groups, and religious services. Four primary themes emerged regarding the relationship between CI/ADRD and social engagement: decreased levels of social engagement, reduced variety of social activities, lowered motivation, and heterogeneity in social engagement. Results suggest that people with CI/ADRD have reduced social engagement, but the evidence is limited due to the inconsistent use of measures for social engagement and CI/ADRD. Future research is needed to develop valid and reliable measures of social engagement that can be used among individuals living with CI/ADRD.

